# Correlation between MRI morphological response patterns and histopathological tumor regression after neoadjuvant endocrine therapy in locally advanced breast cancer: a randomized phase II trial

**DOI:** 10.1007/s10549-021-06343-z

**Published:** 2021-08-06

**Authors:** Joana Reis, Owen Thomas, Maryam Lahooti, Marianne Lyngra, Hossein Schandiz, Joao Boavida, Kjell-Inge Gjesdal, Torill Sauer, Jürgen Geisler, Jonn Terje Geitung

**Affiliations:** 1 Department of Diagnostic Imaging and Intervention, Akershus University Hospital (AHUS), Postboks 1000, 1478 Lørenskog, Norway; 2 Institute of Clinical Medicine, Campus AHUS, University of Oslo, Postboks 1000, 1478 Lørenskog, Norway; 3 Translational Cancer Research Group, Akershus University Hospital (AHUS), Postboks 1000, 1478 Lørenskog, Norway; 4 Health Services Research Department, Akershus University Hospital (AHUS), Postboks 1000, 1478 Lørenskog, Norway; 5 Department of Pathology, Akershus University Hospital (AHUS), Postboks 1000, 1478 Lørenskog, Norway; 6 Sunnmøre MR-Clinic, Agrinorbygget, Langelansveg 15, 6010 Ålesund, Norway; 7 Department of Oncology, Akershus University Hospital (AHUS), Postboks 1000, 1478 Lørenskog, Norway

**Keywords:** Locally advanced breast cancer, MRI, Response patterns, Exemestane, Letrozole, Tumor cellularity

## Abstract

**Purpose:**

To correlate MRI morphological response patterns with histopathological tumor regression grading system based on tumor cellularity in locally advanced breast cancer (LABC)-treated neoadjuvant with third-generation aromatase inhibitors.

**Methods:**

Fifty postmenopausal patients with ER-positive/HER-2-negative LABC treated with neoadjuvant letrozole and exemestane given sequentially in an intra-patient cross-over regimen for at least 4 months with MRI response monitoring at baseline as well as after at least 2 and 4 months on treatment. The MRI morphological response pattern was classified into 6 categories: 0/complete imaging response; I/concentric shrinkage; II/fragmentation; III/diffuse; IV/stable; and V/progressive. Histopathological tumor regression was assessed based on the recommendations from The Royal College of Pathologists regarding tumor cellularity.

**Results:**

Following 2 and 4 months with therapy, the most common MRI pattern was pattern II (24/50 and 21/50, respectively). After 4 months on therapy, the most common histopathological tumor regression grade was grade 3 (21/50). After 4 months an increasing correlation is observed between MRI patterns and histopathology. The overall correlation, between the largest tumor diameter obtained from MRI and histopathology, was moderate and positive (r = 0.50, P-value = 2e-04). Among them, the correlation was highest in type IV (r = 0.53).

**Conclusion:**

The type II MRI pattern “fragmentation” was more frequent in the histopathological responder group; and types I and IV in the non-responder group. Type II pattern showed the best endocrine responsiveness and a relatively moderate correlation between sizes obtained from MRI and histology, whereas type IV pattern indicated endocrine resistance but the strongest correlation between MRI and histology.

**Supplementary Information:**

The online version contains supplementary material available at 10.1007/s10549-021-06343-z.

## Introduction

Chemotherapy is still a major cornerstone of neoadjuvant systemic therapy for patients with locally advanced breast cancer (LABC). However, neoadjuvant endocrine therapy (NET) with third-generation aromatase inhibitors has been considered a low toxicity and valid alternative for either strongly hormone-sensitive tumors (in postmenopausal women and/or for patients not suitable for chemotherapy due to their advanced age or comorbidities); [1–12]. While NET allows the estimation of endocrine responsiveness “in vivo,” an early and more effective prediction whether NET will be effective would benefit patients and enable a better selection and personalization of the treatment, i.e., expedite surgery or switch to neoadjuvant chemotherapy (NAC) in poor responders.

Currently, response monitoring and assessment of residual disease during and after neoadjuvant systemic therapy are conducted with imaging techniques prior to histopathological evaluations. Breast magnetic resonance imaging (MRI) is the most accurate and recommended modality, often considered as the gold standard [1, 13]. However, tumor extent is often more challenging to assess after neoadjuvant therapy and MRI might both over- and underestimate the residual tumor size [14]. There are two main patterns of tumor size response following neoadjuvant systemic therapy: some tumors can show a concentric shrinkage pattern, while others may fragment into scattered islands of tumor cells embedded in connective and fatty tissue [15, 16]. In addition to the effect of NET on tumor size, neoadjuvant systemic therapies often generate a profound effect on tumor cellularity. The overall loss of cellularity after therapy is not always accompanied by a reduction in tumor size, making residual tumor cellularity an important factor in assessing response [17–20].

A previous study by Tozaki et al. concluded that computed tomography (CT) classification of tumor distribution prior to NAC and shrinkage patterns subsequent to NAC is important for the evaluation of the residual disease undergoing breast-conserving surgery [21]. Kim et al. reported MRI response patterns of breast carcinomas and concluded that there is a significant difference in MRI-based response patterns following NAC when comparing histopathological responders and non-responders [22, 23].

**Fig. 1:**
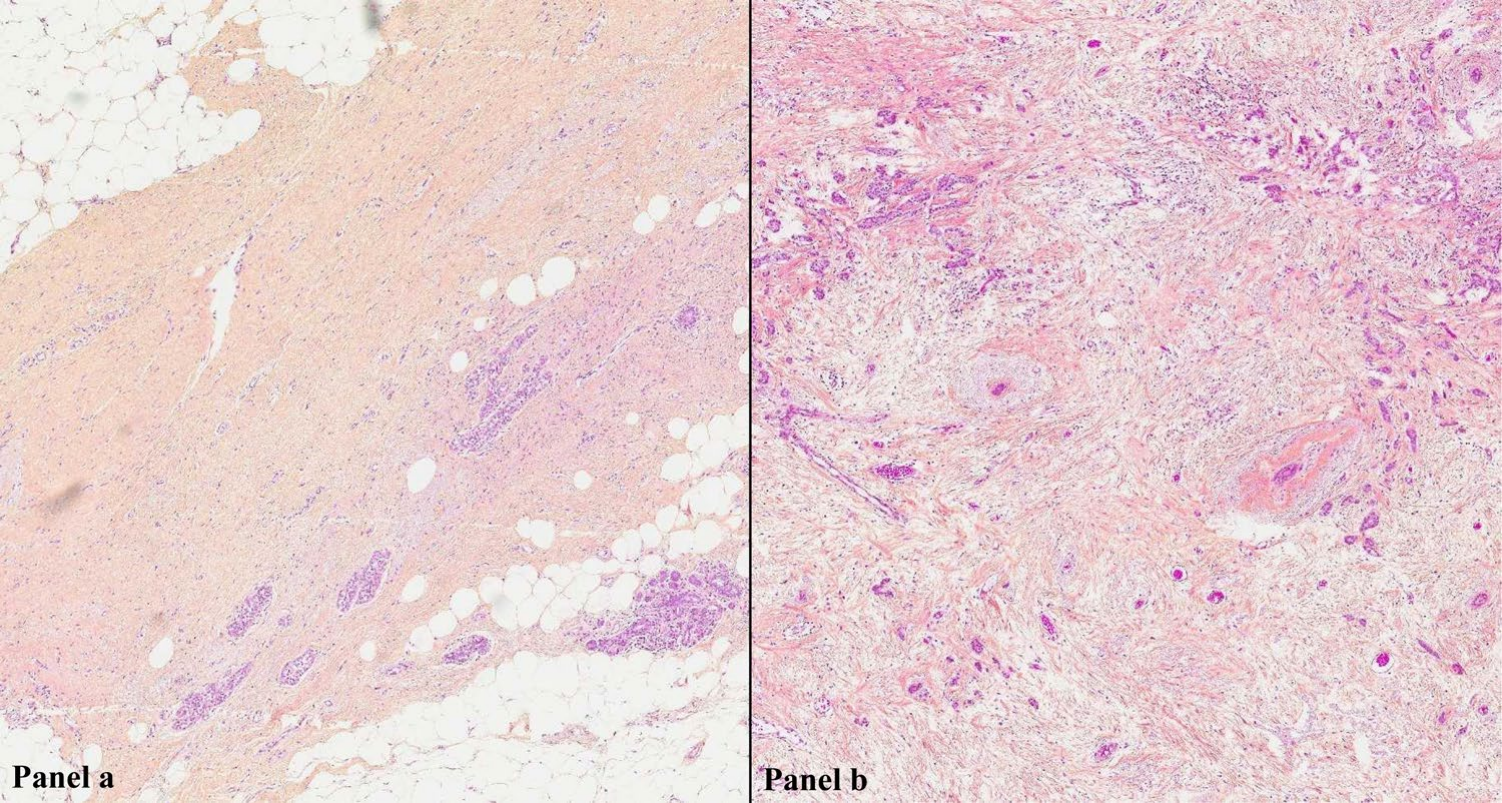
Panoramic view of histopathological tumor regression grades (hematoxylin–eosin-saffron stain, magnification × 400) of ER + /HER2- LABC after 4 months with NET and postsurgery: **a** complete pathological response and **b** moderate partial response to therapy (10–50% of tumor remaining). *ER* estrogen receptor, *HER2* human epidermal growth factor receptor 2; *LABC* locally advanced breast cancer; *NET* neoadjuvant endocrine therapy

**Fig. 2:**
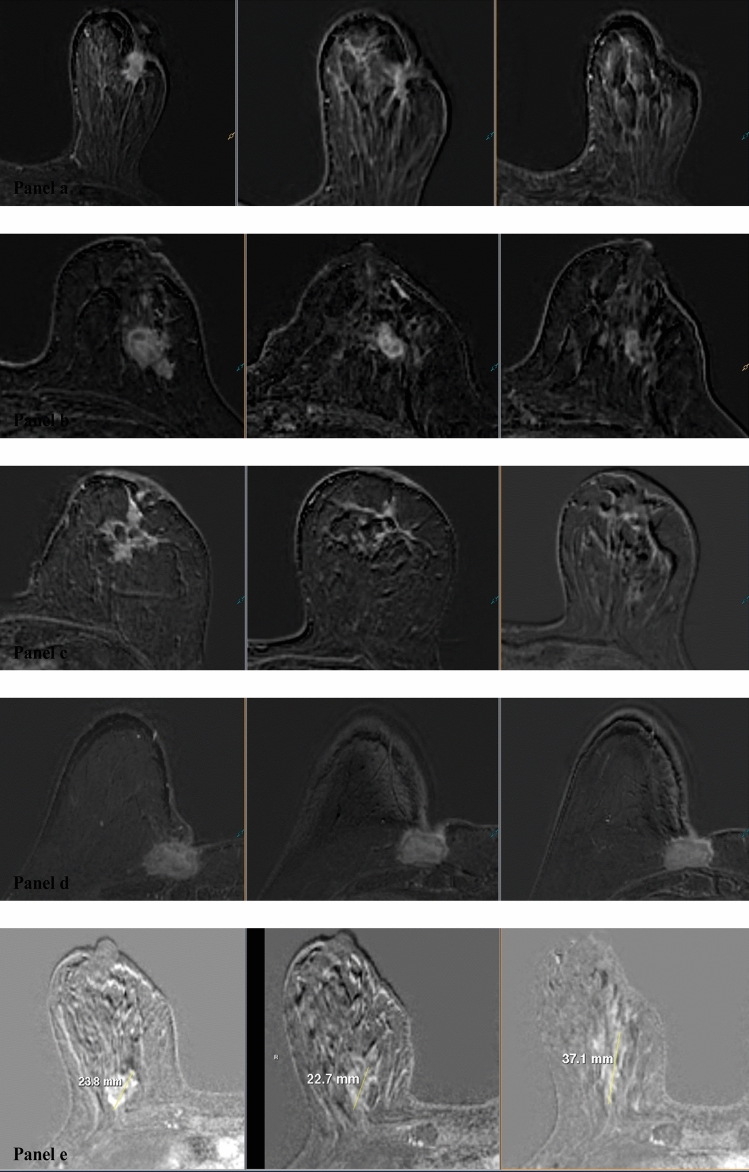
MRI morphological response patterns of ER + /HER2- LABC at three different time points (from left to right): baseline, between regimens (after 2 months of treatment), and presurgery (after 4 months of treatment); **a** a tumor that shrinks concentrically after 2 months with NET, while at the end of therapy, the patient achieved a complete imaging response; **b** a tumor that shows concentric shrinkage without surrounding lesion following 2 and 4 months with NET; **c** a tumor that shows fragmentation pattern throughout the intended therapy; **d** MRI shows a concentric pattern of tumor regression after 2 months, but reveals a stable disease after 4 months with NET, and **e** progressive disease. *ER* estrogen receptor; *HER2* human epidermal growth factor receptor 2; *LABC* locally advanced breast cancer, *MRI* magnetic resonance imaging, *NET* neoadjuvant endocrine therapy

The correlation between MRI morphological response patterns, prior and subsequent to NET with third-generation aromatase inhibitors, and histopathological tumor regression grading system based on tumor cellularity in patients diagnosed with LABC has not been reported. In our study, we evaluate histopathological tumor regression applying a scoring system in compassing five tumor regression grades recommended by The Royal College of Pathologists (Fig. 1), and MRI response patterns (Fig. 2) using 6 categories adapted from the classification suggested by Kim et al. [17, 22].

Hence, we aimed to evaluate whether there is a difference in MRI morphological response patterns between pathological responder and non-responder groups during and after completion of NET. The secondary goal was to compare the largest tumor diameter of histopathology measurements with the largest tumor diameter obtained from MRI according to the MRI morphological response patterns after completion of the intended regimen. The clinical value of our findings is underlined by radiological–pathological correlation, thus validating the implementation of a standardized tumor regression grading system and imaging monitoring for an accurate and prognostic relevant evaluation of tumor response and residual disease after neoadjuvant systemic therapy.

## Methods

This current manuscript is from a substudy of the NEOLETEXE trial. The NEOLETEXE trial was registered on March 23rd, 2015 in the National trial database of Norway and approved by the regional ethical committee of the South-Eastern Health Region in Norway (registration number: REK-SØ-84–2015).

### Patient cohort and treatment

**Fig. 3:**
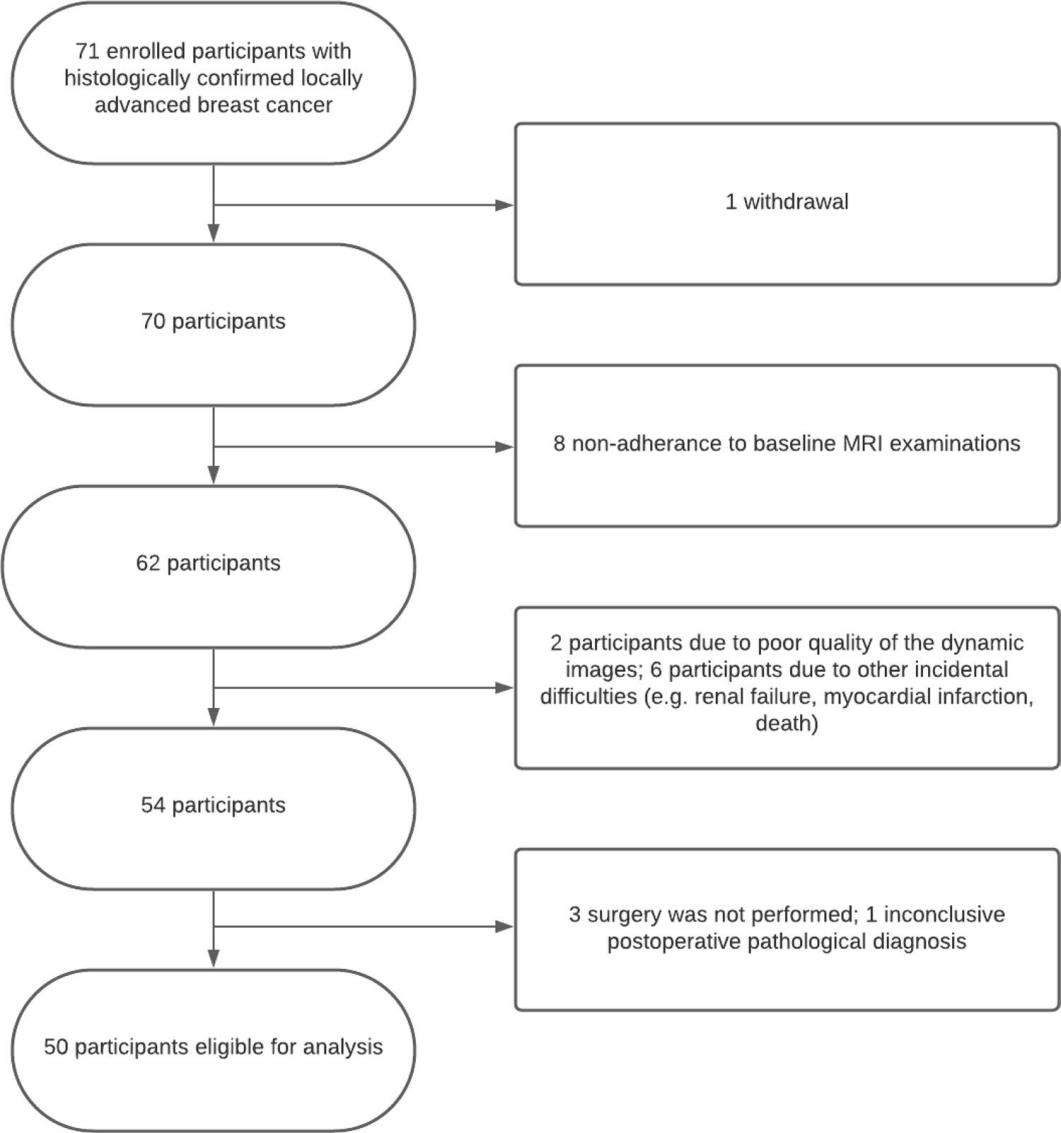
Flowchart of the number of participants eligible for analysis

Our prospective, randomized, open-label, cross-over substudy from the NEOLETEXE trial enrolled 71 participants with histologically confirmed unilateral strongly ER + , human epidermal growth factor receptor 2 (HER2)-negative LABC between February 2015 and December 2020 at Akershus University Hospital (Fig. 3) [24]. The 50 eligible participants had to be postmenopausal to benefit from neoadjuvant aromatase inhibitors (NAAI) with no or limited distant metastasis. The inclusion and exclusion criteria are given in Supplementary † [14]. Patient selection for neoadjuvant treatment was determined by the multidisciplinary breast cancer team at the Akershus University Hospital. The main aim of NEOLETEXE trial is to explore the phenomenon of a lack of cross-resistance between the reversible nonsteroidal imidazole-based inhibitor letrozole (Femar®/Femara®) and the irreversible steroidal activator exemestane (Aromasin®). The exact mechanism and the reason for sequencing two different aromatase inhibitors have been analyzed in a different spinoff biological study [24]. The NAAI intra-patient cross-over regimen consisted of one of the following treatment arms: (1.) letrozole 2.5 mg o.d. for at least 8 weeks thereafter continuing with exemestane 25 mg o.d. for at least another 8 weeks prior to surgery; and (2.) exemestane 25 mg o.d. for at least 8 weeks thereafter continuing with letrozole 2.5 mg o.d. for at least another 8 weeks prior to surgery. Routine and study-specific MRI sequences were performed at baseline and following at least 2 months and 4 months on aromatase inhibitor treatment. Distant metastasis (M stage) was screened with thoracic, abdominal and pelvic CT scan, and bone scintigraphy according to clinical practice. Patient characteristics are summarized in Table 1.

**Table 1:** Study participant characteristics

Parameter	Value	Percentage
No. of eligible participants	50	98
No. of women	49	2
No. of men	1	
Age (y)		
Median	76	
Mean ± standard deviation	74.2 ± 7.3	
Tumor grade		
1 2 3 x	4 37 7 2	8 74 14 4
Tumor histology		
IC-NST ILC Other	36 12 2	72 24 4
cT T2 T3 T4	3 12 35	6 24 70
cN N0 N1 N2 N3	38 9 2 1	76 18 4 2
cM M0 M1 Mx	48 1 1	96 2 2
Surgical treatment		
Breast-conserving surgery Mastectomy	8 42	16 84
ypTN		
T0/Tis/T1/T2/T3/T4/Tx N0/N1/N2/N3	3/1/9/30/5/1/1 23/18/7/2	6/2/18/60/10/2/2 46/36/14/4

The TNM used is the seventh edition. Unless otherwise specified, value column represents the number of eligible patients and mean ± standard deviation, and percentage column shows the percentages of eligible patients, c: clinical staging, *IC-NST* invasive carcinoma of No Special Type, *ILC* invasive lobular carcinoma, *TNM* tumor-node-metastasis, *yp* neoadjuvant pathological staging

### MRI acquisition

Breast MRI was performed on a Philips Ingenia 1.5-T system using a dedicated 16-channel bilateral breast coil with parallel imaging capabilities (Philips Healthcare, Best, the Netherlands).

In addition to breast MRI for staging, the participants underwent two dynamic sequences applied in an interleaved pattern prior and during the injection of the contrast agent. The high temporal resolution images were acquired using a 3D T1_T2*-weighted multi echo-planar imaging sequence and intercalated with a dynamic high spatial resolution 3D T1-weighted turbo field echo sequence (3D T1W THRIVE). Details of the breast MRI sequences have been published previously and are found in Supplementary Table 2 [14]. The institutions breast imaging radiologists interpreted these examinations, according to the American College of Radiology Breast Imaging-Reporting and Data System (BI-RADS®) lexicon (ACR BI-RADS® Atlas 2013, https://www.acr.org/Clinical-Resources/Reporting-and-Data-Systems/Bi-Rads).

## MRI interpretation: baseline enhancement patterns and morphological response patterns

**Fig. 4:**
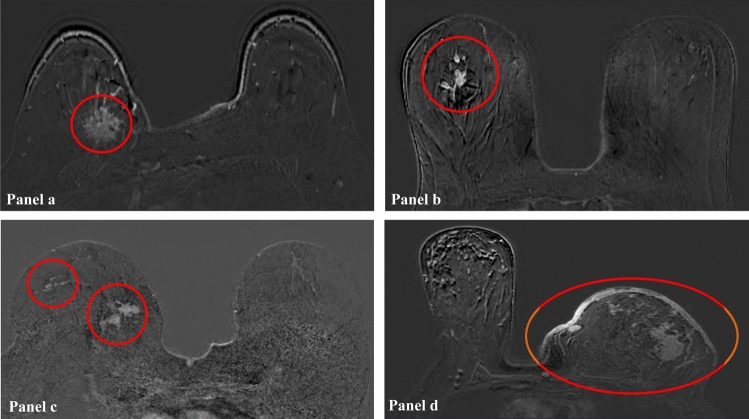
MRI baseline contrast enhancement patterns of ER + /HER2- LABC prior to NET classified into 4 categories: **a** solitary pattern of the right breast, **b** grouped pattern of a localized mass with adjacent spotty enhancement of the right breast, **c** separated pattern of a multicentric LABC of the right breast, and **d** replaced pattern (diffuse contrast enhancement in whole quadrants) of a deformed left breast with thickened skin. *ER* estrogen receptor; *HER2* human epidermal growth factor receptor 2; *LABC* locally advanced breast cancer; *MRI* magnetic resonance imaging; *NET* neoadjuvant endocrine therapy

MRI analysis was done independently by two experienced radiologists (J.R. and M.L.) who were blinded for clinicopathological data. The longest tumor dimension was measured consecutively after imaging acquisition on the 3D T1W THRIVE late peak enhancement sequences and based on the response evaluation criteria in solid tumors (RECIST). The baseline contrast enhancement patterns were classified into 4 categories by modifying Tozaki’s classification [21]: solitary, grouped (localized mass with adjacent linear or spotty enhancement), separated (multifocal or multicentric masses), and replaced (diffuse contrast enhancement in whole quadrants) (Fig. 4). The morphological response patterns were classified into 6 categories adapted from the classification suggested by Kim et al. at between regimens and presurgery time points [22]: 0 (complete imaging response), I (concentric shrinkage), II (fragmentation), III (diffuse contrast enhancement), IV (stable disease), and V (progressive disease) (Fig. 2).

### Histopathological evaluation

The 50 resection specimens were evaluated according to the principles within national and institutional guidelines for standardization of processing and reporting of breast specimens [17]. Histopathological measurement of residual tumor size, which was used as the gold standard, was performed in fresh tissue and correlation was tested macro- and microscopically. Microscopic characteristics of the tumor, including histological tumor type and grade, were recorded, along with lymph node, lymphovascular invasion, and resection margin status. The extent of the tumor was determined applying the standard ypTN (7th edition) restaging system of the largest contiguous focus of invasive cancer (T stage) and the extent of regional lymph node involvement (N stage); yp indicates that participants had received neoadjuvant treatment [16, 25]. Additionally, the assessment of tumor response to treatment was graded based on the recommendations from The Royal College of Pathologists, which has the merit of simplicity and takes account of neoadjuvant therapy-induced tumor cellularity changes: 1 (complete pathological response), 2 (marked partial response to therapy), 3 (moderate partial response to therapy), 4 (minor partial response to therapy), and 5 (no evidence of response to therapy) (Fig. 1). Consequently, divided into 2 groups: pathological responder (1, 2, and 3) and non-responder (4 and 5). The details of histopathological tumor regression grading system can be found on Table 2. Total extent of residual disease was reported, measured as the greatest one-dimensional extent in centimeters of residual invasive cancer including intervening areas of fibrosis and/or necrosis and in situ component. Comprehensive histopathological analysis of surgical specimens was performed separately by experienced breast cancer pathologists (M.L. and H.S.).

**Table 2:** Histopathological tumor regression grading system recommended by the Royal College of Pathologists

Tumor regression grade	Description
1 Complete pathological response	No residual carcinoma or no residual invasive tumor but in situ component may be present
2 Partial response to therapy (marked)	Minimal residual disease/near total effect, e.g., < 10% of tumor remaining in the tumor bed seen as an area of residual fibrosis delineating the original tumor extent
3 Partial response to therapy (moderate)	10–50% of tumor remaining
4 Partial response to therapy (minor)	> 50% of tumor remaining
5 No evidence of response to therapy	No change or minimal alteration to individual malignant cells, but no reduction in overall cellularity

## Statistical analysis

Descriptive statistics were presented using percentages and frequency tables for qualitative variables.

The Cochran–Armitage test for trend (two-sided test) was used to correlate MRI morphological response patterns between responder and non-responder groups and the Kendall rank correlation coefficient to compare different MRI ratings. The largest tumor diameter obtained by MRI after completing treatment was correlated with the largest tumor diameter of histopathology measurements using the Pearson correlation coefficient test (*r*). The agreement between both observers classifying the response according to the six MRI-based patterns after 2 and 4 months with NET was tested with the Kendall rank correlation coefficient. Results were deemed significant at *P* < 0.05. All statistical procedures were performed with R software.

## Results

### Participant characteristics

Of the 71 participants enrolled in this substudy, only 1 withdrew from the study, whereas 8 did not perform the baseline MRI scans as planned (Fig. 3). Two participants were excluded from analysis due to poor quality of the dynamic images. Other incidental difficulties led to the exclusion of another 6 participants (e.g., renal failure, myocardial infarction, death). One patient was excluded because of an inconclusive postoperative pathological diagnosis. Three patients refused surgery and they were subsequently excluded. In total, 50 participants were available for the analysis in this study, the demographics of which are given in Table 1. In brief, the mean age was 74.2 years ± 7.3 (standard deviation), with 49 female and 1 male patient. T stage 2–4 was distributed as 6%, 24%, and 70%, respectively. Seventy-two percent were diagnosed as invasive ductal carcinoma of no special type, 24% were invasive lobular carcinoma, and 4% were classified as invasive papillary and mucinous carcinomas. Eighty-four percent of the patients underwent mastectomy, whereas 16% were scheduled for breast-conserving surgery. The majority of patients underwent axillary lymph node dissection.

### Kendall rank correlation

The Kendall rank correlation coefficient (tau) showed a distinct agreement between observers after 2 months (tau: 0.37 and two-sided *P* value = 0.004) and an even stronger agreement with a very significant *P* value after 4 months (tau: 0.52 and two-sided *P* value = 3e-05); therefore, only one set of results from the two observers (J.R.) is reported in the results section.

## MRI baseline contrast enhancement patterns and MRI morphological response patterns

The most common MRI baseline contrast enhancement pattern was solitary (26 cases), and the second most common pattern was grouped (14 cases); followed by separated (8 cases) and replaced (2 cases). Table 3 summarizes the association between MRI baseline contrast enhancement patterns and MRI morphological response patterns following 2 and 4 months with NET.

**Table 3:** MRI baseline contrast enhancement patterns and MRI morphological response patterns after 2 and 4 months with NET

MRI baseline contrast enhancement pattern	Solitary (n:26)	Grouped (n:14)	Separated (n:8)	Replaced (n:2)
After 2 months				
MRI morphological response pattern				
0 (n:1)	0	0	0	1
I (n:20)	12	6	1	1
II (n:24)	11	8	5	0
III (n:0)	0	0	0	0
IV (n:5)	3	0	2	0
V (n:0)	0	0	0	0
After 4 months				
MRI morphological response pattern				
0 (n:3)	1	0	1	1
I (n:14)	7	5	2	0
II (n:21)	9	8	4	0
III (n:0)	0	0	0	0
IV (n:11)	8	1	1	1
V (n:1)	1	0	0	0

*MRI* magnetic resonance imaging, *NET* neoadjuvant endocrine therapy; *MRI* morphological response patterns: *0* complete imaging response, *I* concentric shrinkage, *II* fragmentation, *III* diffuse contrast enhancement, *IV* stable disease, and *V* progressive disease

After 2 months with NET, the most common MRI morphological response pattern identified was type II (24/50). The second most common pattern identified was type I (20/50). Five cases showed type IV, while only one case showed type 0. The most common MRI morphological response pattern of solitary lesions was type I (12/26, 46.2%), in contrast to grouped and separated lesions, which demonstrated that type II was the most frequent (8/14, 57.1%; and 5/8, 62.5%, respectively). The replaced lesions showed type 0 (1/2, 50%) and type 1 (1/2, 50%).

After 4 months with NET, the most common MRI morphological response pattern identified was type II (21/50), followed in order by type I (14/50), type IV (11/50), type 0 (3/50), and type V (1/50). Nine (34.6%) of 26 solitary lesions showed type II, 8 (57.1%) of 14 grouped lesions showed type II, 4 (50%) of 8 separated lesions showed type II, and 1 (50%) of 2 replaced lesions showed type 0 and the other 1 showed type IV. None of the three patients with complete imaging response after 4 months with NET had grouped baseline contrast enhancement pattern. None of the patients in the present study showed type III MRI response patterns neither after 2 nor 4 months.

### MRI morphological response patterns and histopathological tumor regression grading

Table 4 includes the correlation between histopathological tumor regression grading system and MRI morphological response patterns after 2 and 4 months with NET.

**Table 4:** Correlation between MRI morphological response patterns and histopathological tumor regression grading system after 2 and 4 months with NET

	Histopathological tumor regression grading				
	1 (n:4)	2 (n:4)	3 (n:21)	4 (n:16)	5 (n:5)
After 2 months					
MRI morphological response pattern					
0 (n:1)	0	0	0	1	0
I (n:20)	2	2	8	6	2
II (n:24)	1	2	12	7	2
III (n:0)	0	0	0	0	0
IV (n:5)	1	0	1	2	1
V (n:0)	0	0	0	0	0
After 4 months					
MRI morphological response pattern					
0 (n:3)	1	0	0	2	0
I (n:14)	0	1	6	5	2
II (n:21)	2	2	10	4	3
III (n:0)	0	0	0	0	0
IV (n:11)	1	1	4	5	0
V (n:1)	0	0	1	0	0

*MRI* magnetic resonance imaging, *NET* neoadjuvant endocrine therapy; *MRI* morphological response patterns: *0* complete imaging response, *I* concentric shrinkage, *II* fragmentation, *III* diffuse contrast enhancement, *IV* stable disease, and *V* progressive disease; Tumor regression grades: *1* pathological complete response, *2* < 10% of tumor remaining, *3* 10–50% of tumor remaining, *4* > 50% of tumor remaining, and *5* no reduction in overall cellularity

The most common histopathological tumor regression grade was grade 3 (21/50, 42%), followed by grade 4 (16/50, 32%). In 4 cases showing grade 1 (4/50, 8%), no residual invasive tumor was observed in these cases; in contrast with 5 cases showing grade 5 (5/50, 10%), no reduction in overall cellularity. Four cases showed grade 2 (4/50, 8%). After 2 months, one case was not visualized on MRI, but the lesion was classified as histopathological grade 4 (> 50% of tumor remaining), representing a false-negative case on MRI. Of 3 lesions that showed complete imaging response after 4 months, only 1 was histopathological grade 1, indicating pathological complete response. However, 2 lesions were histopathological grade 4, demonstrating imaging false-negative cases. Histopathological analysis of these 2 false-negative cases was more than one microscopic cluster of invasive lobular cancer cells without mass formation.

After 4 months with therapy, of 21 lesions with type II MRI response pattern, grade 3 was most frequently observed (47.6%), followed by grade 4 (19.0%). Histopathological tumor regression grade 1 was found in 2 cases, pointing to false-positive cases on MRI. Histopathological findings of these two abovementioned cases were focal lobular lymphocytic infiltration with adenosis and fibrous stroma containing numerous foamy histiocytes, respectively. Of 14 lesions with type I imaging response pattern, grade 3 was most frequently observed (43.0%), followed in order by grade 4 (36.0%), grade 5 (14.0%), and grade 2 (7.0%). There was no case of grade 1. Of 11 lesions with MRI response pattern type IV, 5 lesions were grade 4, 4 lesions were grade 3, and 1 lesion was grade 2. There was 1 false-positive case, and histopathological findings were microscopically sparsely scattered foci of ductal carcinoma in situ (DCIS). The 1 lesion showing progressive disease on MRI was histopathological grade 3.

**Fig. 5:**
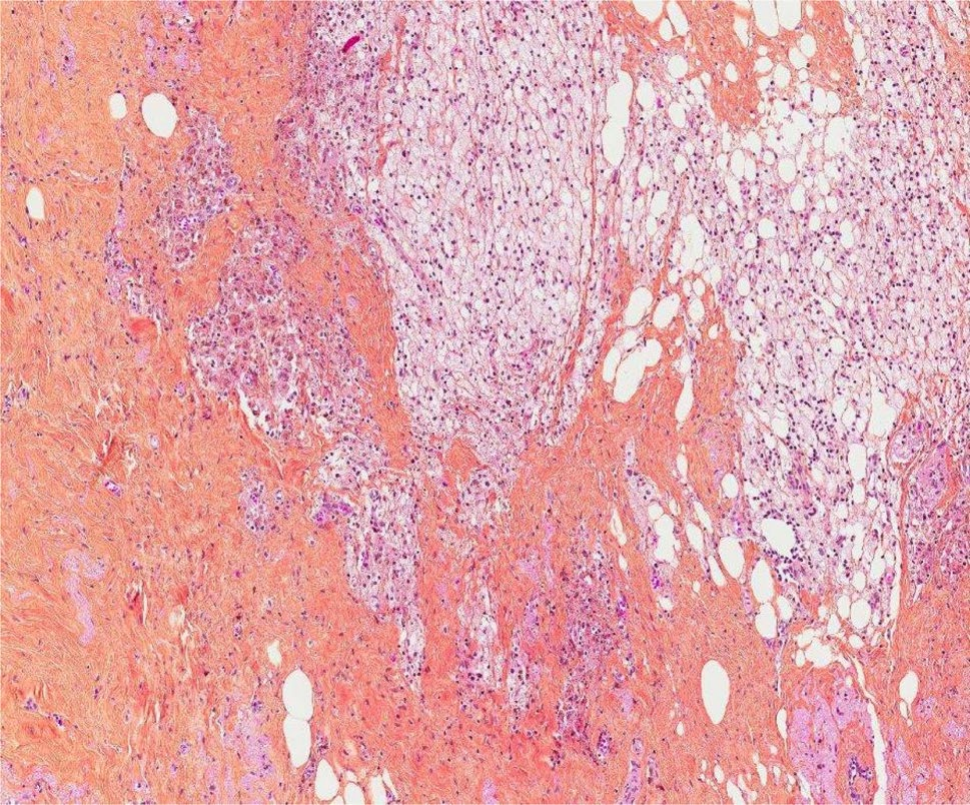
Histopathological findings in LABC after 4 months following completion of NET and postsurgery. On microscopic examination (hematoxylin–eosin-saffron stain, original magnification × 200) there were no residual malignant cells. Tumor bed showed focal area of loose, fibrous, edematous reactive stroma with variable inflammatory cell infiltrate that included collections of lipid and/or hemosiderin-laden macrophages, foamy histiocytes, lymphocytes, and plasma cells. Back-ground breast lobules appear hyalinized and atrophic with a perilobular lymphocytic infiltrate. Microscopically, these features are consistent with complete response to NET. *LABC* locally advanced breast cancer; *NET* neoadjuvant endocrine therapy

**Fig. 6:**
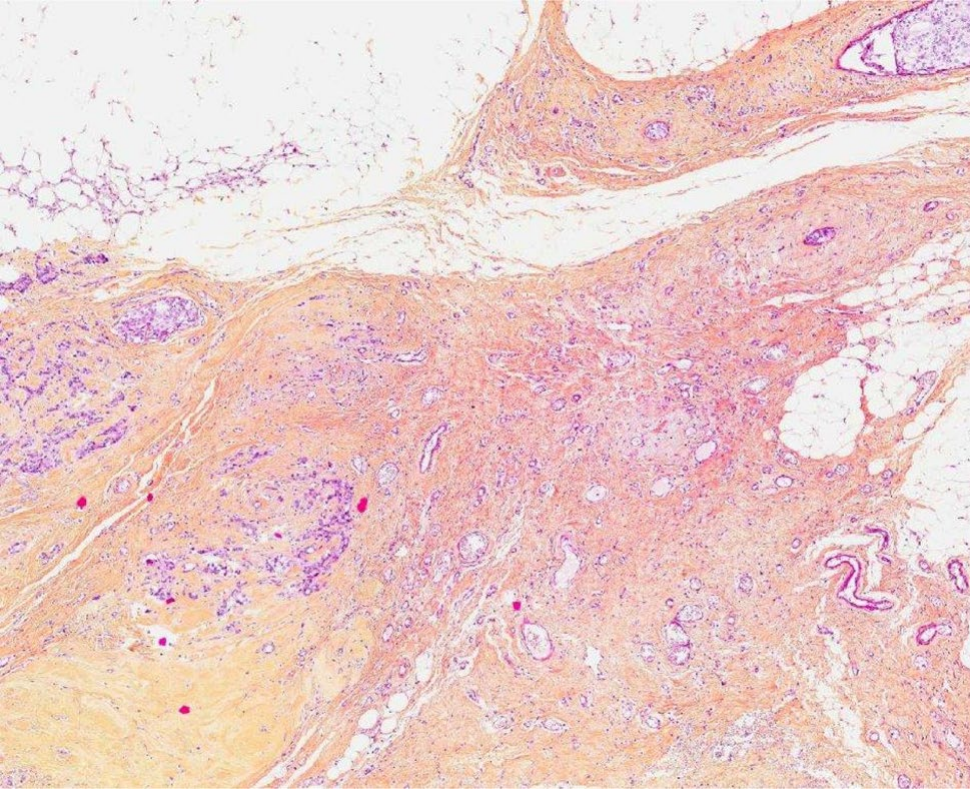
Histopathological findings in LABC after 4 months following completion of NET and postsurgery. Microscopic examination (hematoxylin–eosin-saffron stain, original magnification × 200) showed scar with accumulation of macrophages, hemosiderin deposition, and residual carcinoma cells. Microscopically, these features are consistent with partial response to NET. *LABC* locally advanced breast cancer; *NET* neoadjuvant endocrine therapy

The rate of responder cases (grades 1, 2, and 3) was 58% (29/50) and the rate of non-responder cases (grades 4 and 5) was 42% (21/50) (Figs. 5 and 6). The Kendall rank correlation coefficient indicated absence of association after 2 and 4 months (tau = 0.03 and tau = -0.10, respectively) between MRI patterns and histopathological tumor regression grades. The Cochran–Armitage test determined a slight decreasing trend after 2 months (*Z* = 0.06, dim = 4, *P* value = 0.95), but showed an increasing trend after 4 months between MRI morphological response patterns and responder and non-responder groups (*Z* = 0.98, dim = 5, *P* value = 0.33).

### Histopathological and MRI measurements after 4 months with NET

As a result of a very small number of variables, we decided to exclude from correlation coefficient calculation lesions that showed complete imaging response (pattern 0, 3 cases), diffuse contrast enhancement (pattern III, 0 cases), and progressive disease (pattern V, 1 case) on MRI. When the largest tumor diameter obtained from MRI was correlated with the largest tumor diameter of histopathology measurements, the overall correlation was moderate and positive (*r* = 0.50, *P* value = 2e-04). According to the MRI morphological response pattern, lesions with types I, II, and IV demonstrated positive correlation with the histological diameter (Table 5). Among these types, the correlation coefficient was highest in type IV (*r* = 0.53), followed by type II (*r* = 0.50) and type I (*r* = 0.33).

**Table 5:** Correlation between the largest tumor diameters measured at MRI and histopathology according to the MRI response patterns after 4 months with NET

MRI morphological response pattern	Histopathological diameter median and mean	MRI diameter median and mean	Pearson correlation coefficient (*r*)	*P* value
Observer 1				
I	3, 2.7	3, 3.2	r = 0.33	0.25
II	3, 3.5	3, 3.6	r = 0.50	0.02
IV	3, 2.7	3, 3.1	r = 0.53	0.09

*MRI* magnetic resonance imaging, *NET* neoadjuvant endocrine therapy, *MRI* morphological response patterns: *0* complete imaging response, *I* concentric shrinkage, *II* fragmentation, *III* diffuse contrast enhancement, *IV* stable disease, and *V* progressive disease

### Discussion

Neoadjuvant systemic endocrine therapy is increasingly used in the treatment of LABC for highly selected patient groups to improve patient outcome, avoid side effects of chemotherapy, increase the chance for breast-conserving surgery, and eliminate distant micrometastases [7, 26, 27]. A feasible response monitoring and more accurate assessment of residual disease in the breast and axilla would be clinically of pivotal importance to determine the efficacy of new agents in neoadjuvant clinical trials and to select patients for breast-conserving surgery.

To investigate features for the evaluation of tumor response related to treatment outcome, we explored the MRI morphological response patterns, prior and subsequent to combined NET with third-generation aromatase inhibitors, with histopathological tumor regression grading system based on tumor cellularity in patients diagnosed with ER-positive /HER-2-negative LABC. Secondary, we also compared preoperative MRI largest tumor diameter with largest tumor diameter obtained from the histopathology measurements according to the MRI morphological response patterns. There have been several studies providing criteria for pathological response after NAC, but very limited regarding NET [14, 16, 23].

Our study partly resembled the study of Kim et al., however, adjusting their classification and incorporating a halfway MRI sequence (i.e., after 2 months with therapy) and MRI morphological response pattern IV (stable disease). Kim et al. found that most solitary lesions before NAC showed a concentric shrinkage pattern without adjacent spotty or linear enhancement, which results were similar to ours after 2 months with NET [22]. However, after 4 months in our study, fragmented pattern was most frequently observed. In case of grouped lesions, their results demonstrated that concentric shrinkage with adjacent spotty or linear enhancement was most frequently observed (50%, 9/18), followed by multiple residual nodular pattern (22%, 4/18) and concentric shrinkage without surrounding lesions (17%, 3/18). Our results were analogous in case of grouped lesions, demonstrating that 57.1% (8/14) showed fragmented pattern after 2 and 4 months. In our study, type II pattern (fragmentation) englobes both concentric shrinkage with surrounding lesions and residual multinodular lesions, corresponding to type II and III, respectively, in Kim et al. study. Like their results, concentric shrinking and fragmented tumors were more frequently observed in pathological responder group.

Patients who achieve a complete pathological response of the primary tumor in response to NAC have better prognosis than those who not. The prognosis of patients with partial remission or stable disease is variable, and further refinement of response assessment would be necessary [22]. This strongly highlights the importance of inclusion of stable tumor regression pattern (type IV) in our analysis.

In addition to its effect on tumor size, NET often has a profound effect on tumor cellularity [28–30]. The product of pathological size and tumor cellularity provides more accurate pathological response information than tumor size alone [15, 18, 19, 23, 31]. We used the histopathological tumor regression scoring system that compasses 5 tumor regression grades recommended by The Royal College of Pathologists taking into account the neoadjuvant therapy-induced tumor cellularity changes. [17]. The rate of responder cases (grades 1, 2, and 3) was 58% (29/50) and the rate of non-responder cases (grades 4 and 5) was 42% (21/50). There was no statistically significant difference in response patterns between responder and non-responder groups. This result may be due to our small sample size, but the observed effects might become significant if more data were collected; the different choice of MRI-based response patterns (not including the stable disease pattern in the overmentioned study, they assumed all tumors shrink after therapy, while in our study 22.0% of tumors showed a stable response); and the histopathological tumor regression system used.

Our pooled analysis was also not able to validate the correlation between MRI morphological response patterns and histopathological tumor regression grading system. However, it did point to the increasing trend after 4 months between MRI morphological response patterns and responder and non-responder groups. There are several factors that could have affected the diagnostic accuracy of MRI for therapy response assessment in our study. Tumor molecular subtype is one key factor. Accuracy of MRI in determining residual tumor size after neoadjuvant therapy is greatest in ER-/HER-2 + and triple-negative tumors and is less accurate in luminal tumors [32, 33]. The use of antiangiogenic drugs (such as letrozole and exemestane) could also have influenced diagnostic accuracy of MRI, through hypothesized antivascular effects on contrast enhancement [34]. By targeting the vascular endothelial growth factor (VEGF) and ER signaling pathways simultaneously, aromatase inhibitors may provide a strengthened therapeutic benefit in ER-positive breast cancer. Estrogen-bound ER enhances VEGF expression, providing a common link between these signaling pathways that may be targeted by endocrine therapy and likely contribute to the angiogenic balance in breast cancer patients [35]. Thirdly, the use of pathological response criteria that allow for the presence of noninvasive disease in their definition of complete response can negatively affect the accuracy of imaging response assessment since noninvasive disease may still be visualized with imaging.

We also compared the largest diameter obtained at MRI with the largest histopathological diameter according to the MRI morphological response patterns. According to Wasser et al., the size of tumor with more regressive change was less correlated with histological size compared with the tumor with less regressive change [29]. The correlation was moderate and highest in type IV (stable disease) followed by type II (fragmentation). In cases of type I pattern, the correlation was weak. We would like to stress the following: as in clinical setting, tumors were only measured as the greatest one-dimensional extent in centimeters of residual invasive cancer on MRI and in one cutting direction by the pathologists. If the specimen cutting direction was different from the MRI measurements, the tumor diameter might differ. Additionally, MRI often underestimates or overestimates the extent of the residual tumor following neoadjuvant therapy because of changes in cellularity and/or vascularity. The overall loss of cellularity after NET is not always reflected by a decrease in tumor size, because although tumor cells are destroyed, the host response of reactive inflammation and the fibrous stroma remains, and these fibrotic changes in the breast parenchyma have been demonstrated as persistent enhancement within the treated tumor bed. On the contrary, when the residual cancer cells appear as small foci or scattered cells, they receive nutrients via diffusion and not via vascular perfusion. Thus, it is difficult to detect such residual disease based on MRI contrast enhancements [14, 22, 34]. In 4 patients with complete pathological responses after 2 months on treatment, 2 lesions showed pattern I, 1 lesion showed pattern II, and 1 lesion showed pattern IV, confirming 4 false-positive cases on MRI. After 4 months, one of the 2 lesions that showed pattern I was not visualized on MRI, demonstrating imaging complete response. The other 3 lesions, 2 showed patterns II (one showed invasive lobular cancer cells and the other papillary breast cancer cells) and 1 showed pattern IV. This one showed microscopically sparsely scattered foci of DCIS without an invasive component. Indeed, the presence or absence of residual in situ is another factor that explains the measurement differences.

Our study had some limitations. It was a single-center study with a relatively small sample size. However, MRI data at three distinct time points in 50 patients makes our analysis clinically relevant. We did not evaluate the disease-free and overall survival rates because of the relatively short postsurgery follow-up time. We determined tumor cellularity of histopathological sections from the pretreatment core needle biopsy before NET and from the resection specimen after NET, and there might be some bias. Core needle biopsy specimens could underestimate the overall cellularity at resection [31]. Optimization of our MRI and histopathological protocols might be needed before clinical implementation. Our results are unprecedented and novel, and our findings will require further validation in larger and external cohorts.

To the best of our knowledge, the present study reports the first correlation between MRI morphological response patterns and histopathological residual tumor patterns subsequent to NET.

One major point seems to be that type II was more frequent in the pathological responder group; and types I and IV in the non-responder group. Type II pattern showed the best endocrine responsiveness and a relatively moderate correlation between sizes obtained from MRI and histology. Whereas tumors with type IV pattern demonstrated endocrine resistance but the strongest correlation between sizes obtained by MRI and histology.

Imaging assessment to neoadjuvant endocrine therapy in vivo offers unique opportunities for patient care, research, and clinical decision-making. Clearly, prospective evaluation and monitoring of tumor response with breast high-resolution MRI within a clinical trial setting should routinely incorporate emerging technologies, breast tissue predictive biomarkers, and genetic platforms to allow accurate prediction and assessment of response. Standardized determination of MRI response patterns and histopathological tumor regression models during NET present promising results and provide valuable information that may help to guide surgeons to choose the best type of surgery for an individual patient. The different MRI response patterns suggest the existence of distinct subgroups of luminal A patients that deserve additional investigation to improve the use of response evaluations techniques during NET even further.

## Supplementary Information

Below is the link to the electronic supplementary material.

Supplementary file 1 (DOCX 19 kb)

## Abbreviations

CT: Computed tomography
DCIS: Ductal carcinoma in situ
ER: Estrogen receptor (alpha)
HER-2: Human epidermal growth factor receptor 2
LABC: Locally advanced breast cancer
MRI: Magnetic Resonance Imaging
NAAI: Neoadjuvant aromatase inhibitor
NAC: Neoadjuvant chemotherapy
NET: Neoadjuvant endocrine treatment
o.d.: Once daily
VEGF: Vascular endothelial growth factor

## Declarations

### Availability of data and material

The datasets used and/or analyzed during the current study are available from the corresponding author on reasonable request.

## References

[1] Ragusi MAA, Loo CE, van der Velden BHM, Wesseling J, Linn SC, Beets-Tan RG, Elias SG, Gilhuijs KGA (2020). Contralateral parenchymal enhancement on breast MRI before and during neoadjuvant endocrine therapy in relation to the preoperative endocrine prognostic index. Eur Radiol.

[2] Curigliano G, Burstein HJ, Winer EP, Gnant M, Dubsky P, Loibl S, Colleoni M, Regan MM, Piccart-Gebhart M, Senn HJ (2019). De-escalating and escalating treatments for early-stage breast cancer: the St. Gallen international expert consensus conference on the primary therapy of early breast cancer 2017. Ann Oncol.

[3] Curigliano G, Burstein HJ, Winer EP, Gnant M, Dubsky P, Loibl S, Colleoni M, Regan MM, Piccart-Gebhart M, Senn HJ (2018). De-escalating and escalating treatments for early-stage breast cancer: the St Gallen international expert consensus conference on the primary therapy of early breast cancer 2017. Ann Oncol.

[4] Curigliano G, Burstein HJ, Winer EP, Gnant M, Dubsky P, Loibl S, Colleoni M, Regan MM, Piccart-Gebhart M, Senn HJ (2017). De-escalating and escalating treatments for early-stage breast cancer: the St. Gallen international expert consensus conference on the primary therapy of early breast cancer 2017. Ann Oncol.

[5] Cottu P, D'Hondt V, Dureau S, Lerebours F, Desmoulins I, Heudel PE, Duhoux FP, Levy C, Mouret-Reynier MA, Dalenc F (2018). Letrozole and palbociclib versus chemotherapy as neoadjuvant therapy of high-risk luminal breast cancer. Ann Oncol.

[6] Spring LM, Gupta A, Reynolds KL, Gadd MA, Ellisen LW, Isakoff SJ, Moy B, Bardia A (2016). Neoadjuvant endocrine therapy for estrogen receptor-positive breast cancer: a systematic review and meta-analysis. JAMA Oncol.

[7] Barroso-Sousa R, Silva DD, Alessi JV, Mano MS (2016). Neoadjuvant endocrine therapy in breast cancer: current role and future perspectives. Ecancermedicalscience.

[8] Geisler J, Lonning PE (2005). Aromatase inhibition: translation into a successful therapeutic approach. Clin Cancer Res.

[9] Miller WR (2003). Aromatase inhibitors: mechanism of action and role in the treatment of breast cancer. Semin Oncol.

[10] Wang M, Chen H, Wu K, Ding A, Zhang M, Zhang P (2018). Evaluation of the prognostic stage in the 8th edition of the American Joint Committee on Cancer in locally advanced breast cancer: an analysis based on SEER 18 database. Breast.

[11] Leal F, Liutti VT, Antunes dos Santos VC, Novis de Figueiredo MA, Macedo LT, Rinck Junior JA, Sasse AD (2015). Neoadjuvant endocrine therapy for resectable breast cancer: a systematic review and meta-analysis. Breast.

[12] Madigan LI, Dinh P, Graham JD (2020). Neoadjuvant endocrine therapy in locally advanced estrogen or progesterone receptor-positive breast cancer: determining the optimal endocrine agent and treatment duration in postmenopausal women-a literature review and proposed guidelines. Breast Cancer Res.

[13] Lobbes MB, Prevos R, Smidt M, Tjan-Heijnen VC, van Goethem M, Schipper R, Beets-Tan RG, Wildberger JE (2013). The role of magnetic resonance imaging in assessing residual disease and pathologic complete response in breast cancer patients receiving neoadjuvant chemotherapy: a systematic review. Insights Imaging.

[14] Reis J, Lindstrom JC, Boavida J, Gjesdal KI, Park D, Bahrami N, Seyedzadeh M, Melles WA, Sauer T, Geisler J (2020). Accuracy of breast MRI in patients receiving neoadjuvant endocrine therapy: comprehensive imaging analysis and correlation with clinical and pathological assessments. Breast Cancer Res Treat.

[15] Goorts B, Dreuning KMA, Houwers JB, Kooreman LFS, Boerma EG, Mann RM, Lobbes MBI, Smidt ML (2018). MRI-based response patterns during neoadjuvant chemotherapy can predict pathological (complete) response in patients with breast cancer. Breast Cancer Res.

[16] Provenzano E, Bossuyt V, Viale G, Cameron D, Badve S, Denkert C, MacGrogan G, Penault-Llorca F, Boughey J, Curigliano G (2015). Standardization of pathologic evaluation and reporting of postneoadjuvant specimens in clinical trials of breast cancer: recommendations from an international working group. Mod Pathol.

[17] Pathology reporting of breast disease in surgical excision specimens incorporating the dataset for histological reporting of breast cancer [file:///Users/Joana/Downloads/G148_BreastDataset-hires-Jun16.pdf]

[18] Ballesio L, Gigli S, Di Pastena F, Giraldi G, Manganaro L, Anastasi E, Catalano C (2017). Magnetic resonance imaging tumor regression shrinkage patterns after neoadjuvant chemotherapy in patients with locally advanced breast cancer: correlation with tumor biological subtypes and pathological response after therapy. Tumour Biol.

[19] Choi WJ, Kim WK, Shin HJ, Cha JH, Chae EY, Kim HH (2018). Evaluation of the tumor response after neoadjuvant chemotherapy in breast cancer patients: correlation between dynamic contrast-enhanced magnetic resonance imaging and pathologic tumor cellularity. Clin Breast Cancer.

[20] Yoshikawa K, Ishida M, Kan N, Yanai H, Tsuta K, Sekimoto M, Sugie T (2020). Direct comparison of magnetic resonance imaging and pathological shrinkage patterns of triple-negative breast cancer after neoadjuvant chemotherapy. World J Surg Oncol.

[21] Tozaki M, Kobayashi T, Uno S, Aiba K, Takeyama H, Shioya H, Tabei I, Toriumi Y, Suzuki M, Fukuda K (2006). Breast-conserving surgery after chemotherapy: value of MDCT for determining tumor distribution and shrinkage pattern. AJR Am J Roentgenol.

[22] Kim TH, Kang DK, Yim H, Jung YS, Kim KS, Kang SY (2012). Magnetic resonance imaging patterns of tumor regression after neoadjuvant chemotherapy in breast cancer patients: correlation with pathological response grading system based on tumor cellularity. J Comput Assist Tomogr.

[23] Ogston KN, Miller ID, Payne S, Hutcheon AW, Sarkar TK, Smith I, Schofield A, Heys SD (2003). A new histological grading system to assess response of breast cancers to primary chemotherapy: prognostic significance and survival. Breast.

[24] Bahrami N, Sauer T, Engebretsen S, Aljabri B, Bemanian V, Lindstrom J, Luders T, Kristensen V, Lorentzen A, Loeng M (2019). The NEOLETEXE trial: a neoadjuvant cross-over study exploring the lack of cross resistance between aromatase inhibitors. Future Oncol.

[25] Amin MB, Greene FL, Edge SB, Compton CC, Gershenwald JE, Brookland RK, Meyer L, Gress DM, Byrd DR, Winchester DP (2017). The eighth edition AJCC cancer staging manual: continuing to build a bridge from a population-based to a more "personalized" approach to cancer staging. CA Cancer J Clin.

[26] Ellis MJ, Suman VJ, Hoog J, Lin L, Snider J, Prat A, Parker JS, Luo J, DeSchryver K, Allred DC (2011). Randomized phase II neoadjuvant comparison between letrozole, anastrozole, and exemestane for postmenopausal women with estrogen receptor-rich stage 2 to 3 breast cancer: clinical and biomarker outcomes and predictive value of the baseline PAM50-based intrinsic subtype–ACOSOG Z1031. J Clin Oncol.

[27] Pariser AC, Sedghi T, Soulos PR, Killelea B, Gross CP, Mougalian SS (2019). Utilization, duration, and outcomes of neoadjuvant endocrine therapy in the United States. Breast Cancer Res Treat.

[28] Akashi-Tanaka S, Fukutomi T, Watanabe T, Katsumata N, Nanasawa T, Matsuo K, Miyakawa K, Tsuda H (2001). Accuracy of contrast-enhanced computed tomography in the prediction of residual breast cancer after neoadjuvant chemotherapy. Int J Cancer.

[29] Wasser K, Sinn HP, Fink C, Klein SK, Junkermann H, Ludemann HP, Zuna I, Delorme S (2003). Accuracy of tumor size measurement in breast cancer using MRI is influenced by histological regression induced by neoadjuvant chemotherapy. Eur Radiol.

[30] Yuan Y, Chen XS, Liu SY, Shen KW (2010). Accuracy of MRI in prediction of pathologic complete remission in breast cancer after preoperative therapy: a meta-analysis. AJR Am J Roentgenol.

[31] Rajan R, Poniecka A, Smith TL, Yang Y, Frye D, Pusztai L, Fiterman DJ, Gal-Gombos E, Whitman G, Rouzier R (2004). Change in tumor cellularity of breast carcinoma after neoadjuvant chemotherapy as a variable in the pathologic assessment of response. Cancer.

[32] Mukhtar RA, Yau C, Rosen M, Tandon VJ, I-Spy T, Investigators A, Hylton N, Esserman LJ (2013). Clinically meaningful tumor reduction rates vary by prechemotherapy MRI phenotype and tumor subtype in the I-SPY 1 TRIAL (CALGB 150007/150012; ACRIN 6657). Ann Surg Oncol.

[33] McGuire KP, Toro-Burguete J, Dang H, Young J, Soran A, Zuley M, Bhargava R, Bonaventura M, Johnson R, Ahrendt G (2011). MRI staging after neoadjuvant chemotherapy for breast cancer: does tumor biology affect accuracy?. Ann Surg Oncol.

[34] Fowler AM, Mankoff DA, Joe BN (2017). Imaging neoadjuvant therapy response in breast cancer. Radiology.

[35] Banerjee S, Pancholi S, A'Hern R, Ghazoui Z, Smith IE, Dowsett M, Martin LA (2008). The effects of neoadjuvant anastrozole and tamoxifen on circulating vascular endothelial growth factor and soluble vascular endothelial growth factor receptor 1 in breast cancer. Clin Cancer Res.

